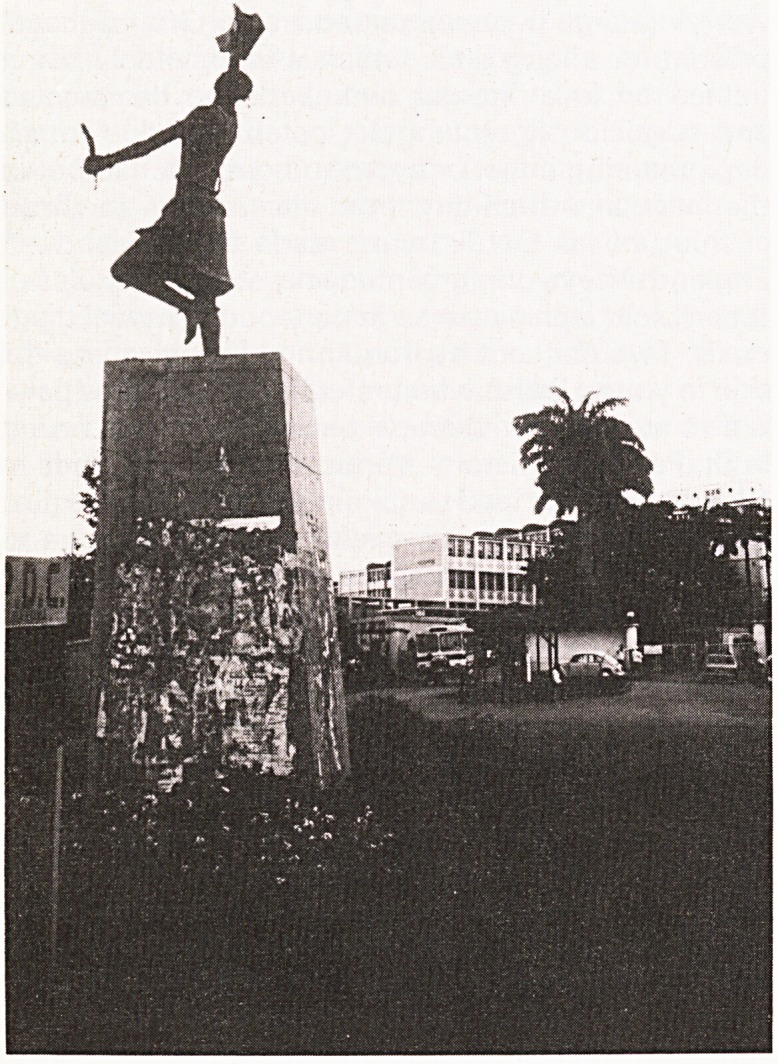# From Our Foreign Correspondent

**Published:** 1983-07

**Authors:** 


### From Our Foreign Correspondent

### A Teaching Visit to the University of Science and Technology, Kumasi, Ghana

A visit to Bristol early last year by the Dean of the School of Medical Sciences, University of Science and Technology (U.S.T.), Kumasi, persuaded me to find the time to spend a month there teaching final year students, and due to the forbearance of my colleagues I was able to set off in October with the minimum of possessions but a large number of teaching slides.

Africa has been a magnet since childhood. A posting to the Army in Nigeria, in the days when National Service gave doctors an opportunity to enlarge their experience abroad, allowed the magnet to function for the first time, and there I was caught and astonished for life. Secondment from St. John's Hospital for Diseases of the Skin when a Senior Registrar to be the first lecturer in Dermatology at University College, Ibadan joined the love of West Africa with my speciality and created lasting links with tropical dermatology, tropical diseases and leprosy. A visiting professorship at Korle Bu Hospital, Accra, Ghana, kept the process going and later work at Nixon Memorial Hospital, Segbwema, some 250 miles inland in Sierra Leone, made the link firmer still. I fancy that I am the only dermatologist in England to have been taught to repair an inguinal hernia by a locally trained theatre master (Mission hospitals in West Africa have theatre masters not sisters).

Ghana's government is at present military, Ghanaian currency is unobtainable in U.K. and there is a curfew from 10.00 p.m. to 5.00 a.m. What one dreads is to arrive in the humid chaos of the airport at Accra in darkness without money and then to find there is no-one to meet you. The worst happened. There was no-one. Plenty of hands anxious to carry my case, heavy with teaching slides, but definitely no smiling face of recognition. But in West Africa you are never alone and must never be unduly put out when plans miscarry. This is normal. A visit to the Swissair office aided by a growing band of helpers and advisers introduced me to the charming wife of the Swissair representative. Her instant reaction to my problem was that I must spend the night at their house and find the U.S.T. guest house in Accra in the morning. The arrival of her husband, however, demolished this plan and I was bought off with an immediate loan of 500 cedi produced from a big leather pouch like those used by Swiss train conductors. With this wealth (about £80 at the non-black market rate) counted out to me in the wide-eyed view of my still interested helpers and feeling particularly vulnerable, a taxi of sorts was found. Off we went into the black night to find the U.S.T. Accra guest house. Ghanaian taxis mostly have a crew of two – one driver and one engineer. The engineer comforted me with the words 'Have no fear, you are not in England, you are in Ghana. We look after you in Ghana.' He told no lies, I *was* looked after and with difficulty, as the notice had long since disappeared from the gatepost, my home for the night was found before the onset of curfew when everybody must be in his home (the University campus at Kumasi, fortunately, counts as 'home').

The norms of life which are carelessly accepted in this country do not apply in Ghana at present. A different set of basics obtains. When one knows what these are likely to be there is no shock but a happy acceptance and a determination to practise the art of the possible. There are telephones but nearly all do not work (not one in operation at the international airport). The airway service between Accra and Kumasi is bookable but mostly unflyable. The main roads may be somewhat maintained in parts but suddenly deteriorate into rocks and laterite holes in others; or they may be unpredictably closed. Petrol is rationed and there are enormous queues of apparently abandoned aged vehicles leading to empty pumps. Extra gallons may be had for a 'consideration', however, and surgeons at least can reverse their cars into the head of the queue, defeat the protesting policeman and come away with a full tank.

The value of money shrinks daily. The minimum daily wage is 12 cedi but a single small meal bought from the lady in the street selling soup and rice costs the poor man 10 cedi. What of his family? Bread is hard to come by because of erratic grain supplies and was not available once during all my time eating at the Senior Common Room, Kumasi. An endless supply of hard and somewhat green ship's biscuit was available, however, and was eatable with a local pattern of margarine, though deadly to crowned teeth. On my last day as a special treat in Accra some 'Ghanaian pure mango preserve' appeared at breakfast specially for me but not at any other time. You do not see overweight people any longer except in an occasional opulent car. Soap is a problem and detergent has disappeared, so the wise visitor takes his own. Electric light bulbs are treasures and toilet rolls priceless. I learned important new practical lessons such as that a visiting doctor in good intestinal health requires one Ghana-made pink toilet roll every 9 days. One 500-g screwtop plastic container of British Daz lasts one man 21 days doing all his own laundry in his bath. One teabag kept for 2 days will make two breakfast cups of tea but not three.

The journey from Accra to Kumasi was made the day after arrival not by plane as was intended but in the good company of the University's finance officer in a Peugeot, none of whose tyres had any tread at all. The doors had to be opened from the outside like a cable car and the windows were not unpleasantly in permanently open positions. This ensured that pale Caucasian features were perfectly tanned with laterite dust on arrival.

The University buildings opened by Prince Philip in 1959 are excellent and imaginative. They sprawl in affluent rural space some 10 miles from Kumasi itself with a huge Ashanti royal stool as an entrance feature. The peace and quiet aspect are a delight.

The guest house for the visitor provides him with perfectly adequate simple accommodation with a balcony and bathroom. The company changes most interestingly at intervals and it is a joy to find a new Ghanaian visiting professor gently easing his morning laundry onto your own private washing line on the balcony as the morning light dawns around 5.30 a.m. Then welcoming him in through the one-time mosquito-proofed doors for a cup of tea and discussion before setting off for the first lecture of the day.

One of my best possessions was a small rucksac without frame which could be folded away easily. In this could be carried projector and slides all ready for lecture to the medical school nearing completion half an hour's walk away. Transport is a constant problem and a bicycle would have been a great asset.

The co-ordinator of the Special Subjects course allocated me a lecture time at 7.15 every morning in the Medical School and it was possible to give a course in dermatology, tropical dermatology and leprosy more extensive than four of us manage in Bristol. Every afternoon there was a period of $1\frac{1}{2}$ to 2 hours for smaller groups of students for practical instruction in talking to patients, history taking, clinical examination, discussion of diagnoses and possibilities of treatment. This was done at the Komfo Anokye Hospital in Kumasi itself 10 miles away, and presented problems. There is no Dermatology Clinic or service in the Hospital though skin disease is almost universal. Patients had to be found by introducing myself to each specialist in his clinic on my second day and showing a willingness to see *any* skin problem daily in a particular teaching room in the afternoon. This method, after a slow and worrying start, produced plenty of common skin problems and a wide variety of general medical complaints but left us short of interesting rarities. It also created a forum for other doctors to bring their 'special' patients to, and a discussion centre which, given time, could have been expanded. Suitable patients could also be found by looking round the crowded wards and finding things of interest. In the paediatric wards this had to be done on the same day as the proposed teaching for children with Kwashiorkor, acute nephroses and toxic epidermal necrolysis,many more than one to a cot, are not necessarily still living next day.

The most punctual person in West Africa was Adu, the driver of the new Sherpa bus allocated to take me to the Hospital after lunch each day. He could be relied on to the minute and the rattle of his diesel engine speeding *round* the speed bumps and up between the teak trees was reassuring. A stone thrown up by a passing wagon had broken his windscreen. You do not find British Leyland windscreens in Kumasi but they can be discovered in Accra. Alas, they had fallen into the hands of agents demanding a 300% mark up and Adu was unable to persuade the University to pay this. So, stalemate, and a steady build-up of laterite dust in the otherwise perfect vehicle discreetly labelled 'Gift from Britain'. Adu became a friend and bought me a fine set of Owari to take home but not at the 300% mark up which would have greeted my face if I had explored the market myself.

The surgeon co-ordinating special subjects had a worrying large hypopigmented area on his forehead, present for a long time, which was inevitably being treated by local steroid ointment. The demarcated and suspiciously rectangular patch was due to the depigmenting effect of hydroquinone leached out in the heat and humidity from the rubber forehead cushion of his E.N.T. mirror made by Gowlands in England. The hypopigmented anaesthetic macules of tuberculoid leprosy are an important differential diagnosis. Two per cent hydroquinone for bleaching the skin in young ladies who prefer their faces to be paler is also available in identical packaging to that found in St. Paul's in Bristol – 'Venus de Milo' and made in Nigeria. This can lead to staining of the nails and also after prolonged use irreversible granular damage to the facial skin. The rare sebaceous gland anomaly of steatocystoma multiplex giving a leonine facies, partial loss of eyebrows and erroneously treated as lepromatous leprosy was also met just as it had been in Shiraz, Iran.

Kumasi is the second city of Ghana and in the heart of Ashanti country. The story is told that it was chosen as the capital due to the flourishing of a Kum tree at that site (Kum = Kum tree, asi = under the) rather than at Kumawu (awu = dead) nearby. The Hospital, now used as the teaching hospital, was originally Kumasi Central Hospital and Nurses' Training Centre with E. H. Adams, A.R.I.B.A., as architect, and Gee, Walker and Slater Ltd. of London as contractors. It is now named after the fetish priest Komfo Anokye. It is finely conceived but the present economic plight of the country makes parts of it a sad sight, and working in it an uphill struggle. Asking for example for a wisp of cotton wool to test for light touch reveals that there is none to be had. Outside under the flame trees there are the derelict remains of what was once a replica of the sword embedded in stone which only the great Komfo Anokye could withdraw. It is strange that our King Arthur legend should repeat itself so far away. At the Hospital entrance is a recent statue of Komfo Anokye clutching a golden stool as it descends from heaven to solve the problems of the local warring kings, each with his own black stool of authority, and to unite them in friendship (see photograph).

But the purpose of my visit, supported by the British Council, was to teach fifth year medical students, and what of them? There were 30, 21 men and 9 women. They live on the campus in fine halls of residence and are the cream of those students destined for university. They are the most responsive and delightful students to teach and be friends with I have ever met. At our early morning lecture as an opening with a grin I would sometimes say 'Well, what have you had for breakfast this morning?' The answer would be water, gari and beans or cerelac which is a sort of fou-fou made from maize. But a consistently neater, more carefully turned out group with pressed clothes, perfectly clean hands and tidy hair, good health and vigour was never seen. We had not a moment's awkwardness at any time. Note taking was careful, attention remarkable and I detected no absences, except one afternoon when I had misunderstood a politely-given message and found they had all gone to collect their monthly allowance. They had my sympathy.

The girls dressed with great care and on a visit to Kokofu Leprosarium, which I was able to organise one Saturday as an extra treat, I was cut to the heart to hear one of the young women patients in an isolation hut with Dapsone resistant lepromatous leprosy asking that she might have a dress like one of the students too.

There is no doubt of the quality of students, the response to a short term visitor and of the yawning gap which he fills, with the possibility later of a visit for some of them to our own hospitals arranged personally. The links between our country with its traditions and expertise are more important than ever and must never be allowed to lapse because of destructive monetarism.

After the last successful coup of 31st December 1981 the universities were closed for four months and the students directed to go to work on the farms. They did, and have survived to achieve the present excellent standard. Possibly they have learned to value their privileged status all the more and know how fragile it is.

The School of Medical Studies was opened at U.S.T. in 1975 and this is the first group of students to complete all their studies at Kumasi. Previous intakes had to go to Korle Bu in Accra for their clinical work. The voyage of the School has been a stormy one and a previous Dean found himself a defendant in court, the charge being dereliction of duty, and the plaintiffs the senior medical students. I found such a situation unthinkable with my group. They work hard and have little outlet for broadening their experience outside the campus. The working day begins at 7.15 a.m. Three lectures have been attended by 10.30 a.m. and the frustrating journey from campus to hospital made. A Special Department Clinic follows before a poor lunch and, while I was there, an afternoon of clinical instruction devoted entirely to them without the Doctor being responsible for looking after large numbers of patients at the same time, as we have to do in Bristol.

Signs of decay at the University are too frequent. For leisure the tennis courts only are in reasonable order (Dr. Emmanuel Asare, B.Sc., M.B., Ch.B. lent me all I needed – shirt, shorts, shoes and socks – the day after I arrived so that I could play three times a week). The Olympic sized swimming pool contains water but is not in use as the filter mechanism is out of order and alum, chlorine and copper sulphate are unobtainable. The magnificent stadium with its notice 'Walking across the stadium is prohibited' is now grazed by cattle. A further notice board on it, with broken glass, still announces 'West African Universities Sports 1973'.

The snooker table in the Senior Common Room, which gets plenty of use, is badly worn. When the Ghanaian Professor of Surgery makes a secure pot on it he exclaims 'As safe as the Bank of England', which has a special meaning in that economy.

In the Library, books are badly out of date. The Medical Register stopped in 1963, Colliers Encyclopaedia is dated 1952 and amongst the dottier 'Gifts from Britain' is the Sewage Works Register for the United Kingdom 1963.

Charming events take place. On opening the guest house front door onto the cool of the early morning with rucksac and projector on back there is the night watchman, Julius, sitting with a torn piece of brown paper and pencil. 'Good morning, Julius, what are you doing?' 'Counting to ninety, Sa'. 'Oh! Let me look, please.' And on sitting down on the concrete with him I see that there are columns of figures on the brown paper arranged in groups of nine instead of ten. This does make counting to ninety much more difficult and he is delighted to be shown the way into the decimal system.

Further on towards the classroom, watching the grey parrots and listening to the frogs in the swamp, there is a tall young man also walking under the shade of the trees with a large skein of grey wool getting into a tangle round his neck as he winds it into a ball. Enormous white knitting needles stick out from a string bag dangling from his waist.

On arriving for lunch one day in something of a hurry I find the usual simple arrangements are disordered by a most important visit of financial officers being entertained by the Vice-Chancellor himself. He immediately invites me to share in the occasion, but before letting me sit down my kind steward, John, who keeps a very special eye on me since I gave him my cigarette ration, takes me aside and whispers 'the trouser are not well closed, Sa'. There is time to correct the error before sitting next to the Vice-Chancellor.

Incongruities abound. When I protest to John that there is no margarine this Friday morning to go with the ship's biscuit he returns from the kitchen with the message 'Cook say maybe Monday'. Cook can then be heard loudly whistling 'The First Nowell'. As I walk to the lecture room one morning pondering a talk on Yaws and Syphilis I wonder about the wisdom of describing snail track ulcers in West Africa. Almost at once on the damp path there is a snail with its track in front of my feet and I know that it will be all right.

Suddenly while sitting outside in the starlit night with Orion very low on the horizon listening to the chattering of bats, the hoot of owls, the croaking of frogs and clicking of crickets, the tropical night is shattered by 'Rule Britannia' and 'God Save the Queen' at full orchestral blast from an African bungalow. The driving is atrocious and to travel at anything less than a dangerously high speed seems to be impolite. As a passing, overloaded truck leaves you in a hail of stones and cloud of dust your cursing of the driver is mitigated by the notice on the tailgate 'Jesus loves you'. Over a hundred miles from the Komfo Anokye Hospital in a University guest house there are some large round wooden packing cases with a remarkable pink dust all round them, clearly having rested there for a very long time. A closer look shows they are labelled 'Calamine B.P. 1968. Made in China.' Who had ordered it? Why was it there and would it ever be put to good use? At the airport at the very moment of arrival on my last day I was assailed by several large women with collecting tins on their heads and all trying to pin flowers into me – it is Poppy Day and, of course, I should have known it happened in November in Ghana too.

The birdlife is wonderful and independent of the economic climate. The visitor should always take a pocket sized pair of binoculars. Elwood's 'Birds of West African Town and Garden' is an excellent start. Black kites, common vultures, pied crows, allied hornbills almost shaking themselves off upper branches as they get more and more excited, Senegal kingfishers on telegraph lines, little African swifts, laughing doves, herons, cattle egrets and African pied wagtails are all easily seen. Also tiny bee eaters, grey parrots, incredible pintailed whydahs, orange cheeked waxbills, common drongos and bul-buls. These last, with their untidy crowns, make the call 'Quick-doctor-quick', but some are slicker in Kumasi and say distinctly 'Quick-doc-quick'.

The value of a short-term lecturer to the School of Medical Sciences can be very great, of a doctor on longer term contract greater still. In our fortunate society there must be many who have expertise to offer and who would enjoy the work. The School is growing, the students are the most hard working and likeable I have ever met (some are coming to Bristol for their elective this year), and the experience as an antidote to the sense of frustration in our contracting Health Service is exhilarating. It should be tried.

*R. R. M. Harman*

## Figures and Tables

**Figure f1:**